# Presentation at the Royal Society of Medicine

**Published:** 1919-07-19

**Authors:** 


### Presentation at the Royal Society of Medicine.

What must have been a very gratifying ceremony to its central figure was enacted in the Barnes Hall of the Royal Society of Medicine on Friday, the 11th inst., in the presence of a large and distinguished gathering, when that cultured and affectionately genial leader of medical thought, Sir William Osler, Regius Professor of Medicine, Oxford, had his seventieth birthday anniversary signalised by the handsome present of seven hundred volumes. The chair was occupied by his brother Regius of Cambridge, Sir Clifford Allbutt, and he performed the ritual on behalf of the participants. Among his prominent supporters were Sir D'Arcy Power, Sir Wm. Hale White, Sir Donald Macalister (President of the General Medical Council), Sir Wilmot Herringham, Sir Anderson Critchett, Sir George Savage. Lady Osler was also present. Sir Norman Moore, Sir Humphry Rolleston, and Sir Fredk. Mott were unable to be present.

**The Chairman** said: Sir William Osler, Ladies and Gentlemen,—To me, as one of your oldest friends in time, and perhaps the oldest in age, has fallen the honour of announcing our celebration of your seventieth birthday, one universal of many years of supreme service in two kindred nations and for the world. The last lustrum of your threescore and ten, if now merged in victory, has been a time of war and desolation, of broken peoples and stricken homes. Yet through this clamour and destruction your voice, among the voices in the serener air of faith and truth, has not failed, nor your labour for the sufferings of others grown weary.

But, while thus we celebrate your leadership in the relief of sickness and adversity, we are far from forgetting the sunnier theme, the debt, none the less, which we owe to you in other fields of thought. In you we see the fruitfulness of the marriage of science and letters and the long inheritance of a culture which, amid the manifold forms of life and through many a winter and summer, has revived to inspire and adorn a civilisation which, so lately, has narrowly escaped the fury of a barbarian.

**Sir William Osler's Seventieth Birthday**—(*cont.*).

And now I will not avoid a topical allusion, an allusion to your recent Presidential Address to the Classical Association at Oxford : an address which, in its various learning, its wisdom and its wit, brilliantly illustrated this fecundity of letters and science, embodied a common spirit of science and art, and conferred a distinction upon our profession.

In these volumes, we hope, you will find the kind of offering from your fellow-workers which will please you best : immaterial offerings indeed, but such as may outlive a more material gift. As to you, we owe much of the inspiration of these essays, and as in many of their subjects you have taken a bountiful part, so, by them, we desire to give some form to our common interests and affections. We pray that health and strength may long be spared to you, and to her who is the partner of your life : and that for many years to come you will abide in your place as a Nestor of modern Oxford, as a leader in the van of medicine, and as an example to us all. (Applause.)

Sir Clifford Allbutt then made the presentation.

**Sir William Osler,** in reply, said : Sir Clifford Allbutt, Ladies and Gentlemen,—As the possessor of a wild-wagging tongue, which has often got me into trouble, I thought it would be better, on such an occasion, to put down what I am going to say. (Laughter.) Two circumstances deepen the pride a man may justly feel at this demonstration of affection by his colleagues on both sides of the Atlantic—one, amid so much mental and physical tribulation my friends should have had the courage to undertake this heavy task. The other is, to receive this presbyonic honour at the hands of my brother Regius, friend of more than forty years. There is no sound more pleasing than one's own praises, but surely an added pleasure is given to an occasion which praises the honourer as much as the honoured. To you, Sir, more than to anyone in our generation, has been given a rare privilege : when young, the old listened to you as eagerly as do now when old the young. (Applause.) Like Hai ben Zagzan, of Avicenna's allegory, you have wrought deliverance to all who have consorted with you.

To have enshrined your gracious wishes in two goodly volumes appeals strongly to one the love of whose life has been given equally to books and men. A glance at the long list of contributors, so scattered over the world, recalls my vagrant career—Toronto, Montreal, London, Berlin, and Vienna as a student; Montreal, Philadelphia, Baltimore, and Oxford as a teacher. Many cities and many men; truly, with Ulysses, I may say : " I am part of all that I have met."

Uppermost in my mind are feelings of gratitude that my lot has been cast in such pleasant places and in such glorious days so full of achievement and so full of promise for the future. Paraphrasing my lifelong mentor—Sir Thomas Browne—among multiplied acknowledgments I can lift up one hand to Heaven that I was born of honest parents, that modesty, humility, patience, and veracity lay in the same egg and came into the world with me. To have had a happy home, in which unselfishness reigned, parents whose self-sacrifice remains a blessed memory, with brothers and sisters helpful far beyond the usual measure—all these make a picture delightful to look back upon. Then to have had the benediction of friendship follow one like a shadow, to have always had the sense of comradeship in work, without the petty pin-pricks of jealousy and controversy, to be able to rehearse in the sessions of sweet silent thought the experiences of long years without a single bitter memory—to do this fills the heart with gratitude. That three transplantations have been borne successfully is a witness to the brotherly care with which you have tended me. Loving our profession, and believing ardently in its future, I have been content to live in it and for it. The moving ambition to become a good teacher and a sound clinician was fostered by opportunities of exceptional character, and any success I may have attained must be attributed, in large part, to the unceasing kindness of colleagues and to a long series of devoted pupils whose success in life is my special pride.

And to a larger circle of men with whom my contact has been through the written word—general practitioners of the English-speaking world—I should like to say how deeply their loyal support has been appreciated. And if, in this great struggle through which we have passed, sorrow came where she had not been before, the blow has been softened by the loving sympathies of many dear friends. And may I add the thanks of one who has loved and worked for our profession, the sweet influences of whose home have been felt by successive generations of students?

To the Committee and Editors I am deeply indebted for the trouble they have taken in these hard days, and to the publisher, Mr. Hoeber, for his really pre-war bravery. And our special thanks are due to you, dear friends—and in this I include Lady Osler's—you who have graced this happy ceremony with your kindly presence. (Prolonged applause.)

**Sir D'Arcy Power:** Ladies and Gentlemen,—It is my very pleasant duty to close this interesting meeting by proposing a vote of thanks to Sir Clifford Allbutt for having come here this afternoon to preside over us and make this presentation. I suppose this is the first occasion on which the two Regius Professors of the two Universities of Oxford and Cambridge have come together for such a function. I hope it is a good augury for the future. We owe to Sir Clifford Allbutt our very best thanks for coming to London for this purpose.

**Sir Donald Macalister:** On behalf of those who are not members of the Committee, for whom Sir D'Arcy Power has spoken, I have been asked to support this vote of thanks to Sir Clifford Allbutt. The function he has carried out is one we all feel grateful for. And, more than that, those of us who come from Cambridge feel proud of him. We were perfectly aware that when the Regius Professor of Cambridge undertook to make the presentation to the Regius Professor of Oxford it would be done in the most perfect possible manner. The —to use his own words—variety of learning, the wit, the wisdom, and, I may add, the deep feeling, which characterised his utterance in making the presentation to our dear friend Osler, fully justified our expectation and our pride. It was only right that Oxford and Cambridge should join in the presentation, and that Oxford and Cambridge should join in thanks to him who has taken the chair. (Applause.)

**Sir Clifford Allbutt:** Ladies and Gentlemen,—That you should so cordially and kindly thank me for taking the chair on this occasion came to me, when a few minutes ago I heard of it, with great surprise. It seems to me contrary to what ought to have taken place, for it is I who ought to thank you for giving me the one great privilege of my life, of coming forward on an occasion which may, perhaps, be described as unique, to voice your feelings in this matter, to be your intermediary in this presentation to our honoured friend Sir William Osler. It is a matter on which I have most cordially to thank you, rather than to receive your thanks.

The proceedings then terminated.

